# Phylogenetic Relationships of Five Asian Schilbid Genera Including *Clupisoma* (Siluriformes: Schilbeidae)

**DOI:** 10.1371/journal.pone.0145675

**Published:** 2016-01-11

**Authors:** Jing Wang, Bin Lu, Ruiguang Zan, Jing Chai, Wei Ma, Wei Jin, Rongyao Duan, Jing Luo, Robert W. Murphy, Heng Xiao, Ziming Chen

**Affiliations:** 1 Yunnan University / State Key Laboratory for Conservation and Utilization of Bio-Resources in Yunnan, Kunming 650091, China; 2 School of Life Science, Yunnan University, Kunming 650091, China; 3 State Key Laboratory of Genetic Resources and Evolution, Kunming Institute of Zoology, Chinese Academy of Sciences, Kunming, Yunnan 650223, China; 4 Centre for Biodiversity and Conservation Biology, Royal Ontario Museum, 100 Queen’s park, Toronto, M5S 2C6, Canada; University of York, UNITED KINGDOM

## Abstract

The phylogenetic relationships of Asian schilbid catfishes of the genera *Clupisoma*, *Ailia*, *Horabagrus*, *Laides* and *Pseudeutropius* are poorly understood, especially those of *Clupisoma*. Herein, we reconstruct the phylogeny of 38 species of catfishes belonging to 28 genera and 14 families using the concatenated mitochondrial genes *COI*, *cytb*, and 16S rRNA, as well as the nuclear genes *RAG1* and *RAG2*. The resulting phylogenetic trees consistently place *Clupisoma* as the sister taxon of *Laides*, and the five representative Asian schilbid genera form two monophyletic groups with the relationships (*Ailia* (*Laides*, *Clupisoma*)) and (*Horabagrus*, *Pseudeutropius*). The so-called “Big Asia” lineage relates distantly to African schilbids. Independent analyses of the mitochondrial and nuclear DNA data yield differing trees for the two Asian schilbid groups. Analyses of the mitochondrial gene data support a sister-group relationship for (*Ailia* (*Laides*, *Clupisoma*)) and the Sisoroidea and a sister-taxon association of (*Horabagrus*, *Pseudeutropius*) and the Bagridae. In contrast, analyses of the combined nuclear data indicate (*Ailia* (*Laides*, *Clupisoma*)) to be the sister group to (*Horabagrus*, *Pseudeutropius*). Our results indicate that the Horabagridae, recognized by some authors as consisting of *Horabagrus*, *Pseudeutropius* and *Clupisoma* does not include the latter genus. We formally erect a new family, Ailiidae fam. nov. for a monophyletic Asian group comprised of the genera *Ailia*, *Laides* and *Clupisoma*.

## Introduction

The family Schilbeidae, one of more than 30 extant families of catfishes, contains five African genera including the type genus *Schilbe* and five Asian genera, including *Clupisoma*, *Platytropius* and *Horabagrus* [1, 2]. Several morphological phylogenetic studies of the Siluriformes, including those of Mo (1991) [3], De Pinna (1993) [4] and Diogo *et al*. (2004) [5], evaluated representative genera of Schibidae. Notwithstanding, the phylogenetic relationships of *Clupisoma* remain unclear because studies other than Mo (1991) [3] did not include both *Clupisoma* and *Platytropius*. Mo examined *Clupisoma* and mentioned *Platytropius*, but failed to comment on the phylogenetic position of *Clupisoma* and did not specify which species of *Platytropius* were examined. Uncertainty exists as to the grouping of genera and the relationship of the Schilbeidae to other catfish families. Molecular phylogenetic analyses by Peng *et al*. (2005) (based on mitochondrial DNA cytochrome b gene sequences) [6], Hardman (2005) (also using cytochrome b) [7] and Sullivan *et al*. (2006, 2008) (by using nuclear genes *RAG1* and *RAG2*) [8, 9] all indicated that the Schilbeidae was not monophyletic, and that the analyzed African genera formed a distantly related monophyletic group. The phylogenetic relationships of the five Asian schilbid genera remain uncertain largely due to variation among studies in taxa included, and incomplete sampling of the Asian genera *Clupisoma*, *Pseudeutropius*, *Ailia*, *Laides* and *Horabagrus*.

Huang (1981) [10] assigned the species *Platytropius sinensis* to *Platytropius*, which originally contained *P*. *siamensis* only [11]. Subsequently this species was placed in *Clupisoma* as *C*. *sinensis* by Ng (1999) [12]. Afterwards, Chen *et al*. (2005) [13] described the new schilbid species *Clupisoma nujiangense* from China while considering *C*. *sinensis* and *C*. *longianalis* to be congeners.

Species of *Clupisoma* are important food catfishes that inhabit the Mekong and upper Salween rivers. In the last two decades, their populations have declined due to over-fishing and anthropogenic habitat changes. Knowledge of the level of genetic diversity of a species can contribute to the understanding of its evolutionary history, and such data are critical for developing effective conservation and management strategies [14]. Genetic diversity may influence the ability of a species to adapt to environmental changes. Thus, such diversity is an important factor in the conservation of endangered species [15].

Herein, we investigate the phylogenetic history of the family Schilbeidae while including representative species of all five Asian genera. Our analyses use the mitochondrial genes *COI*, *cytb*, and 16S rRNA, as well as the nuclear genes *RAG1* and *RAG2*. We aim to resolve the groupings of the Asian genera with the inclusion of the Chinese species *Clupisoma sinensis*.

## Materials and Methods

### Ethics

All the samples of fishes were bought from local fish dealers in Manzha Market in Menglun Town, Mengla County, Yunnan province, China. (21°56′07.30″N,101°14′56.54″E; elevation: 546m). As food fishes, no permits were required for sampling. All the samples were living in the natural body of water. The housing and husbandry conditions were unclear and all fishes were dead when obtained. Specimens were preserved using 70% ethanol in the Laboratory for Conservation and Utilization of Bio-resources, Yunnan University. All procedures followed corresponding regulations and by-laws and were approved by the Ethics and Experimental Animal Committee of Kunming Institute of Zoology, Chinese Academy of Science, China (KIZ_YP201002).

### Sampling and outgroup selection

Seventeen individuals belonging to eight species of six catfish families were sampled (Table 1). Twenty-eight additional sequences from 28 species of 23 genera in 13 catfish families were downloaded from GenBank (Table 1). We used two species each from the Cypriniformes, Clupeiformes and Characiformes as outgroup taxa.

**Table 1:** The species used in this study and GenBank accession numbers.

Families name	Genera name	Scientific name	Locality	COI	16S	Cytb	rag1(1,2)	rag1(3)	rag2
Sisoridae	*Glyptothrax*	*Glyptothrax lampris 1*	China, Yunnan	JN020065	JN020051	JN020080	JN020106	JN020091	JN020122
		*Glyptothrax lampris 2*	China, Yunnan	JN020066	JN020052	JN020081	JN020107	JN020092	JN020123
		*Glyptothrax laosensis*	China, Yunnan	JN020067	JN020053	JN020082	JN020108	JN020093	JN020124
		*Glyptothrax macromaculatus 1*	China, Yunnan	JN020068	JN020054	JN020083	JN020109	JN020094	JN020125
		*Glyptothrax macromaculatus 2*	China, Yunnan	JN020069	JN020055	JN020084	JN020110	JN020095	JN020126
		*Glyptothrax macromaculatus 3*	China, Yunnan	JN020070	JN020054	JN020085	JN020111	JN020095	JN020127
		Bagarius yarrelli*	Thailand	EU417766	AY445910	DQ119406	DQ492552	DQ492446	DQ492334
Pangasiidae	*Pangasius*	*Pangasius beani 1*	China, Yunnan, Menglun	JN020072	JN020057	JN020086	JN020112	JN020097	JN020129
		*Pangasius beani 2*	China, Yunnan, Menglun	JN020073	JN020058	JN020087	JN020113	JN020098	JN020130
	*Helicophagus*	Helicophagus waandersii*	Thailand	\	DQ334328	DQ119468	DQ492585	DQ492515	DQ492402
	*Pangasianodon*	Pangasianodon hypophthalmus*	Thailand, Nonthabur fish	EF609427	GU324167	GQ856796	DQ492637	DQ492517	DQ492404
Siluridae	*Wallago*	*Wallago attu 1*	Yunnan	JN020076	JN020061	AF477828	JN020115	JN020100	JN020133
		*Wallago attu 2*	Yunnan	JN020076	JN020061	AF477828	JN020116	JN020101	JN020134
		*Wallago attu 3*	Yunnan	JN020076	JN020061	AF477828	JN020117	JN020102	JN020135
	*Kryptopterus*	Kryptopterus minor*	Asia, Aquarium fish trade	\	AY458879	AY458895	DQ492600	DQ492486	DQ492373
Cranoglanidae	*Cranoglanis*	Cranoglanis bouderius*	China, Guangxi	AY898626	AY898626	AY898626	DQ492572	DQ492514	DQ492401
Amblycipitidae	*Liobagrus*	Liobagrus anguillicauda*	China	EU490878	AY574353	AF416888	EU490965	EU490983	EU491002
		Liobagrus marginatoides*	China	EU490880	AY445892	EU490929	EU490966	EU490985	EU491005
		Liobagrus marginatus*	China	EU490882	\	EU490930	EU490969	EU490987	EU491006
		Liobagrus sp.*	China	EU490886	\	EU490935	EU490973	EU490990	EU491011
Schilbidae	*Clupisoma* ☆	*Clupisoma sinensis 1*	China, Yunnan, Menglun	JN020077	JN020062	JN020088	JN020118	JN020103	JN020136
		*Clupisoma sinensis 2*	China, Yunnan, Menglun	JN020078	JN020063	JN020089	JN020119	JN020104	JN020137
		*Clupisoma sinensis 3*	China, Yunnan, Menglun	JN020079	JN020064	JN020090	JN020120	JN020105	JN020138
	*Pareutropius*	Pareutropius debauwi*	Rep. Congo	NC015837	NC015837	NC015837	DQ492632	DQ492507	DQ492394
	*Schilbe*	Schilbe intermedius*	Rep. Congo	HM882935	\	AJ245638	DQ492615	DQ492508	DQ492395
*Ailia*+*Laides*	*Ailia*	*Ailia coila*		JN628886	GQ411080	EU490901	DQ492541	DQ492452	DQ492340
	*Laides*	*Laides hexanema*		EU490866	\	EU490915	DQ492601	DQ492453	DQ492341
Horabagidae#	*Horabagrus*	Horabagrus brachysoma*	India	EU490864	HM579855	EU490913	DQ492593	DQ492554	DQ492342
	*Pseudeutropius*	Pseudeutropius brachypopterus*	Sumatra, Batang Hari basin	EU490871	\	EU490920	DQ492624	DQ492455	DQ492343
Clariidae	*Clarias*	*Clarias fuscus*	China, Yunnan	JN020071	JN020056	AF416885	JN020121	JN020096	JN020128
		Clarias batrachus*	Thailand, Chao Phraya basin	EF609334	GQ402540	DQ119486	DQ492568	DQ492521	DQ492408
		Clarias gabonensis*	Gabon	HM882915	\	\	DQ492569	DQ492519	DQ492406
Akysidae	*Acrochordonichthys*	Acrochordonichthys rugosus*	Thailand	DQ508027	\	EU490899	DQ492539	DQ492444	DQ492332
	*Akysis*	Akysis sp.*	Thailand	EU490853	\	EU490902	DQ492542	DQ492445	DQ492333
		Akysis parshadi*	China	EU490854	\	EU490903	EU490960	EU490978	EU490998
	*Breitensteinia*	Breitensteinia cessator*	China	EU490851	\	EU490900	EU490959	EU490977	DQ508040
Bagridae	*Mystus*	*Mystus nemurus 1*	Yunnan	JN020074	JN020059	AF499600*	JN020114	JN020099	JN020131
		*Mystus nemurus 2*	Yunnan	JN020075	JN020060	AF499600*	JN020114	JN020099	JN020132
		Mystus bocourti*	Thailand	EU490863	JQ248058	EU490912	DQ492589	DQ492462	DQ492350
	*Hemibagrus*	Hemibagrus wyckioides*	Thailand, Mekong basin	EU490862	JQ248063	EU490911	DQ492587	DQ492461	DQ492349
	*Leiocassis*	Leiocassis poecilopterus*	Sumatra, Batang Hari basin	EU490867	\	EU490916	DQ492603	DQ492457	DQ492345
Anchariidae	*Gogo*	Gogo arcuatus*	Madagasear, Andriam bombo River	\	FJ013191	FJ0131601	DQ492582	DQ492528	DQ492415
Ariidae	*Cephalocassis*	Cephalocassis borneensis*	Thailand, Chao Phraya basin	\	FJ626071	FJ626200	DQ492563	DQ192525	DQ492412
	*Bagre*	Bagre marinus*	USA	GU225559	DQ990627	AJ581355	DQ492553	DQ492524	DQ492411
Ictaluridae	*Noturus*	Noturus insignis*	USA, NewYork	JN027812	AY458875	AY327303	DQ492639	DQ492513	DQ492400
	*Pylodictis*	Pylodictis olivaris*	USA, Pennsylyania	EU525113	AY458871	AF484161	DQ492619	DQ492512	DQ492399
Heteropneustidae	*Heteropneustes*	Heteropneustes fossilis*	Aquarium fish trade	HQ009491	FJ432687	DQ119383	DQ492591	DQ492522	DQ492409
Characiformes	*Leporinus*	*Leporinus fasciatus*		\	HQ17132	HQ289610	\	HQ289223	HQ289417
	*Piabina*	*Piabina argentea*		HM405183	HQ171283	GU908175	\	HQ289187	HQ289380
Cypriniformes	*Cyprinus*	Cyprinus carpio*		NC001606	NC001606	NC001606	AY787040	AY787040	AY787041
	*Danio*	Danio rerio*		NC002333	NC002333	NC002333	NM131389	NM131389	U71094
Clupeifromes	*Alosa*	Alosa sapidissima*		NC014690	NC014690	NC014690	\	DQ912116	DQ912150
	*Clupea*	Clupea pallasii*		AP009134	AP009134	AP009134	\	DQ912118	DQ912152

*, Sequences derived from GenBank; \, sequences not derived from GenBank

#, reference to the classification of the De Pinna (1993)

☆ Reference to the classification of the Ng (1999) [12] and Chen *et al*. (2005) [13].

### DNA Extraction, PCR and Sequencing

Primers were either designed based on sequences of Pangasiidae retrieved from GenBank by using Primer Premier 5.0 software (Premier Biosoft International), or they were adapted from literature (Table 2). Genomic DNA was isolated from tissue samples by standard phenol/chloroform extraction. PCR were performed in a 30μl reaction mixture containing 20–50 ng templates DNA, 1.2μM dNTP, 0.5μM of the forward and reverse primers, 0.15 units of EX-*Taq* DNA polymerase enzymes (TaKaRa) and 3 μl of 10× EX-*Taq* buffer. The amplification reaction was performed using 33 cycles of 30sec at 95°C, annealing at 66 to 55°C for 30sec, and extension of 72°C for 90sec, with an initial step of 4min at 95°C and a final step of 7min at 72°C. PCR products were purified on agarose gels and extracted (Watson BioMedical Inc. Shanghai) and sequenced with a BigDye DNA sequencing kit (ABI) on a 3730XL sequencer (ABI). The sequences were deposited in GenBank (accession numbers listed in Table 1).

**Table 2:** The primers for PCR amplification and sequencing.

Gene Fragment		Primer sequences (5'→3')		Source
*COI*	F1	TGT AAA ACG ACG GCC AGT ATT CAA CCA ATC ATA AAG ATA TTG G	amplification	Ivanova(2007)
	R1	CAG GAA ACA GCT ATG ACT AAA CTT CTG GAT GTC CAA AAA ATC A		
	F1d	TGT AAA ACG ACG GCC AGT TCT CAA CCA ACC ACA ARG AYA TYG G		
	R1d	CAG GAA ACA GCT ATG ACT AGA CTT CTG GGT GGC CRA ARA AYC A		
	M13F	TGT AAA ACG ACG GCC AGT	Sequencing	Ivanova(2007)
	M13R	CAG GAA ACA GCT ATG AC		
*16S*	R	CGC CTG TTT AAC AAA AAC AT	amplification and Sequencing	Palumbi (1991)
	F	CCG GTC TGA ACT CAG ATC ATG T		
*Cytb*	L14724	GAC TTG AAA AAC CAC CGT TG	amplification	Xiao (2001)
	H15915	CTC CGA TCT CCG GAT TAC AAG AC		
	L15138	ATR ATR ACC GCC TCC GTY GGY TA	Sequencing	Xiao (2001)
	L15519	GGA GAC CCA GAA AAC TTY ACY CC		
	H15287	AGT GGA AGT CGA AGA ATC GTG		
	H15560	GCR TAG GCA AAY AGG AAR TAT C		
*Rag1(5')*	U69	TGT TYC TGG CAG CAT TAT GAA	amplification	
	L1410	TGY TTC TGM GCC CTT CGT		
	U558	CTT CTA GRT GGC CTG AYG T	Sequencing	
	U989	GAW TTY CCA AAA GAY TTT G		
	L594	TTA AAY ACK TTK AGG ATG ACR T		
	L1018	AAT KGC ACT RAC AAA RTC TTT T		
*Rag1(3')*	U47	TTC TTC CKG GST TCC ATC AAT TTG A	amplification	
	L1423	TGT TYC CAG ATT CRT TCC CT		
	U492	GTG YCT CAT GTT YGT GGA T	Sequencing	
	U903	TGC CTT GCA CTG TGA CAT TGG CA		
	L501	CAT GAG RCA CAG WGG CCT RC		
	L928	CAT TGC CAA TRT CAC AGT GC		
*Rag2*	mhf1	TGY TAT CTC CCA CCT CTG CGY TAC C	Amplification and Sequencing	Hardman (2004)
	mhr1	TCA TCC TCC TCA TCK TCC TCW TTG TA		

The PCR amplification primers and sequencing primers of the nuclear genes were designed based on RAG sequences of Pangasiidae in GenBank.

### Sequence analysis

*De novo* sequences were checked using BLAST [20] against the NCBI database (http://www.ncbi.nlm.nih.gov) to assess sequence similarity. They were aligned using ClustalX 1.83 and manually verified. DAMBE 4.1.19 [16] was used to identify unique haplotypes.

### Phylogeny construction

Phylogenies were constructed using maximum likelihood (ML) via RAxML [17], Bayesian inference (BI) executed with MrBaves 3.2 [18], and maximum parsimony (MP) implemented in PAUP* 4.0b10 [19]. We selected the best-fitting models for ML and BI using the Akaike Information Criterion (AIC) [20, 21] as implemented in jModelTest 0.1.1 [22, 23]. BI analysis used four independent MCMC chains run simultaneously for 5 million generations while sampling one tree per 500 replicates, Burnin = 0, and Burninfrac = 0.10, 0.20, 0.30, 0.40, and 0.50. Two runs were conducted independently and the sampled trees were used to construct a 50% majority rule consensus tree after discarding the first 10% as burnin. Bayesian posterior probabilities (BPP), the frequencies of nodal resolution, were mapped on the BI tree. For MP and ML, nodal support was assessed using nonparametric bootstrap sampling [24] of 1000 pseudoreplicates.

### Testing tree incongruence

The incongruence among different tree topologies was evaluated using the Approximately Unbiased (AU) test [25], as implemented in the CONSELV0.1i with default scaling and replicate values [26]. Site-wise log-likelihood values were estimated by PAUP*.

## Results

### MtDNA

The concatenated mtDNA dataset comprised 2300 aligned sites: 626 from the *COI* fragment, 1137 from *cytb*, and 537 from the 16S rRNA fragment. The genes consisted of 41 unique haplotypes for 43 sequences of *COI*, 43 unique haplotypes among 46 sequences of *cytb*, and 33 unique haplotypes among 36 sequences of 16S rRNA. The combined alignment comprised 2300 positions, of which 941 (40.9%) were potentially parsimony-informative (Table 3).

**Table 3:** Summary statistics for the genes used in this study.

	*COI*	*16S*	*cytb*	*RAG1(exon1*,*2)*	*RAG1(exon3)*	*RAG2*
Aligned sites	626	537	1137	1430	1375	945
A% (average)	25.6	31.4	28.6	29.8	26.9	25.6
G% (average)	18.7	22.6	13.9	22.3	26.3	24.7
C% (average)	27.1	24.3	29.1	22.7	22.1	25.7
T% (average)	28.6	21.7	28.5	25.1	24.7	24.5
Variable sites	272 (43%)	206 (39%)	589 (52%)	948 (66%)	675 (49%)	576 (61%)
Parsimony-informative sites	245 (39%)	160 (30%)	536 (47%)	795 (56%)	577 (42%)	472 (50%)

**Fig 1:**
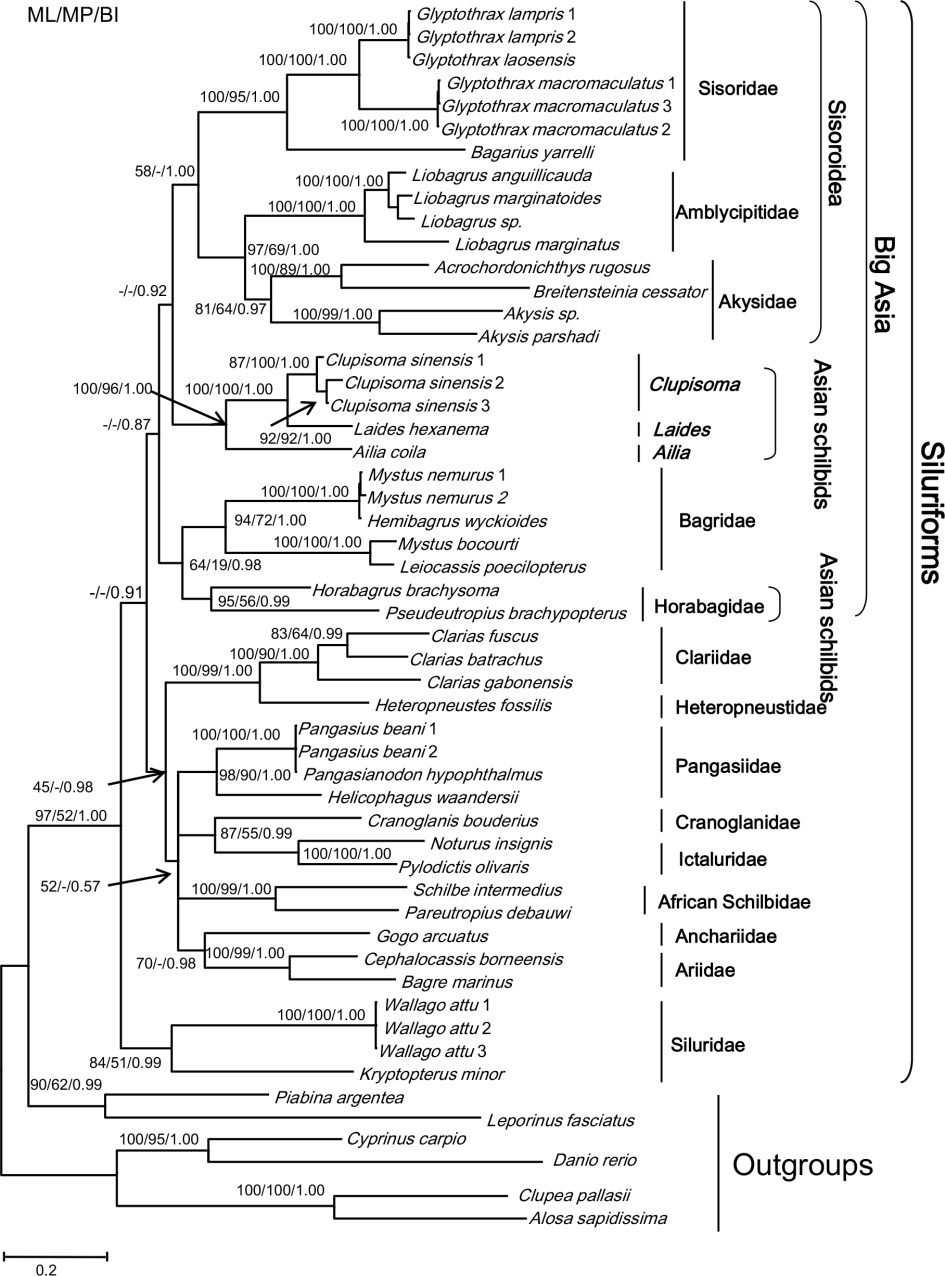
The matrilineal genealogy of the Chinese *Clupisoma* (as *Platytropius*) (Schilbeidae) and *Pseudeutropius* (Pangasiidae) in the Siluriformes derived from the combined mtDNA datasets using ML, MP and BI methods. Nodal support values are indicated on the branches. The names Sisoroidea and “Big Asia” are after Sullivan *et al*. (2006) [8].

Individual mtDNA gene analyses produced inconsistent topologies with low levels of nodal support, probably due to limited information harbored in a single gene. The trees constructed by analyses of the concatenated data using ML, MP and BI (Fig 1) were consistent for well supported nodes. The five Asian schilbid genera formed two monophyletic groups, one consisting of *Clupisoma*, *Lades* and *Ailia* (BI BPP = 1.0, ML BS = 100% and MP BS = 96%) and the other comprising *Horabagrus* and *Pseudeutropius* (BI BPP = 0.99, ML BS = 95% and MP BS = 56%). *Clupisoma* formed the sister taxon of *Laides* (BI BPP = 1.0, ML BS = 100% and MP BS = 100%). Excluding the MP tree, the two Asian schilbid groups rooted within the Bagridae. The superfamily Sisoridae, excluding the Aspredinidae, constituted a lineage referred to as “Big Asia” by Sullivan *et al*. (2006, 2008) [8, 9]. Within “Big Asia”, (*Ailia* (*Laides*, *Clupisoma*)) was the sister-group of the Sisoroidea (BI = 92%), while (*Horabagrus*, *Pseudeutropius*) was the sister taxon of the Bagridae (BI = 98%) (Fig 1).

### NuDNA

**Fig 2:**
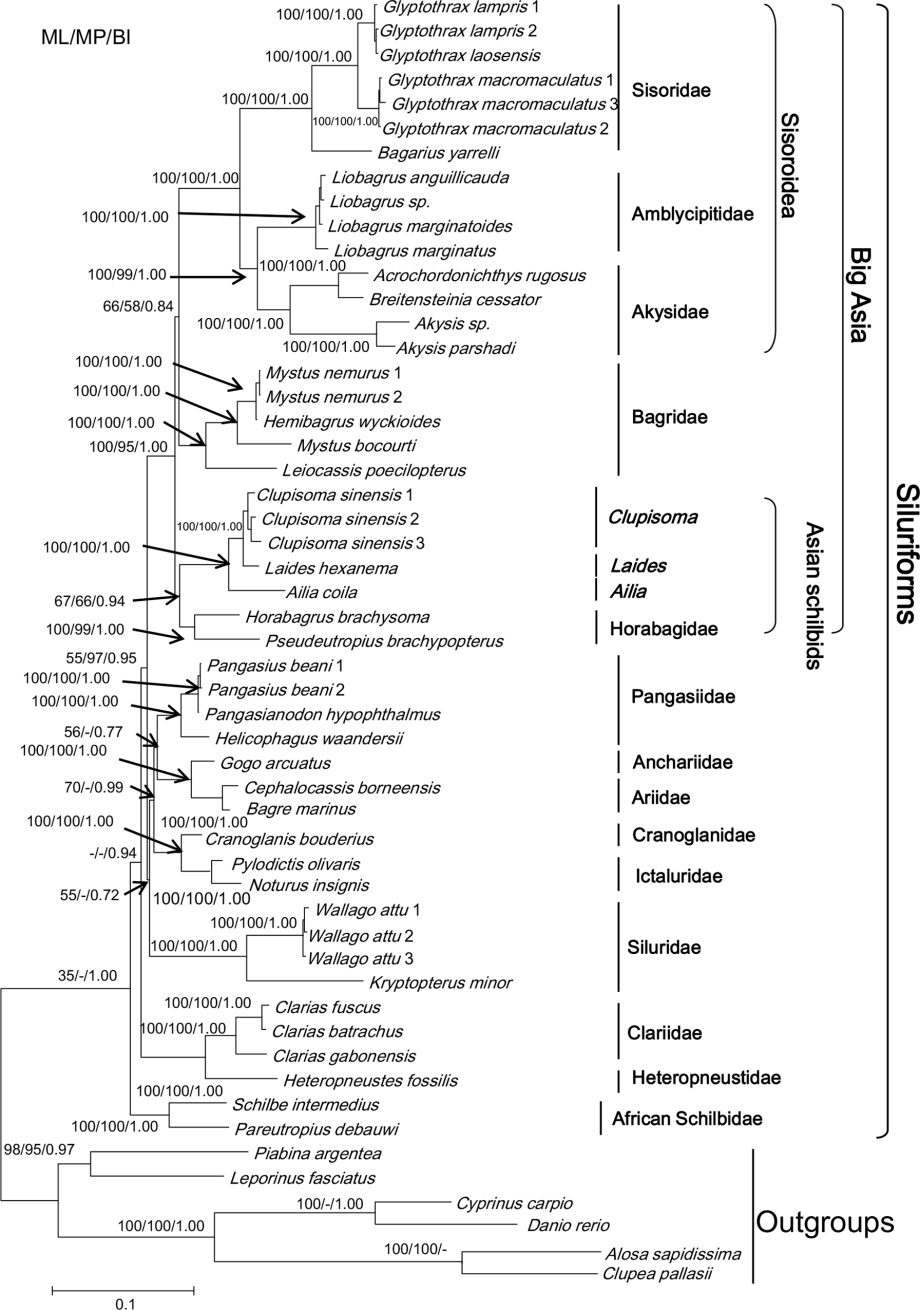
Phylogenetic relationships of the Siluriformes based on ML, MP and BI analysis of the concatenated datasets of nuclear genes. Nodal support values are indicated on the branch. The names Sisoroidea and “Big Asia” are after Sullivan *et al*. (2006) [8].

The combined alignment of the nuclear genes *RAG1* and *RAG2* contained 3750 positions: 1430 from the *RAG1* exon 1, 2), 1375 from the *RAG1* exon 3, and 945 from *RAG2* (Table 3). Among these, 1844 sites (49.3%) were potentially parsimony-informative (Table 3). The tree (Fig 2) displayed likelihood bootstrap proportions, parsimony bootstrap proportions and Bayesian posterior probabilities (BPP).

As with the mt-genes tree, the five Asian schilbid genera also showed the strongly supported monophyletic groups (*Ailia* (*Laides*, *Clupisoma*)) (BI BPP = 1.0, ML BS = 100% and MP BS = 100%) and (*Horabagrus*, *Pseutropius*) (BI BPP = 1.0, ML BS = 100% and MP = 99%). However, analyses of the nuDNA data consistently united them as sister taxa (BI = 94%, ML = 67% and MP = 66%) and rooted them in “Big Asia” with strong support (BI BPP = 1.0, ML BS = 100% and MP = 95%). Relationships among this group, the Bagridae, and the superfamily Sisoroidea were not well resolved.

### Concatenated MtDNA and NuDNA

For a total evidence analysis, we have combined three mtDNA genes (*COI*, 16s and *cytb*) and two nuclear genes (*RAG*1 and *RAG*2). The three mtDNA fragments comprised 2300 aligned sites: 626 from the *COI* fragment, 537 from the 16S fragment, and 1137 from *cytb*; and the nuclear dataset consists of 3750 aligned bases: 1430 from the *RAG1* (exon 1, 2) fragment, 1375 from the *RAG1* (exon 3) fragment and 945 from *RAG*2 (Table 3). The concatenated datasets were comprised of six fragments including 6050 aligned sites.

**Fig 3:**
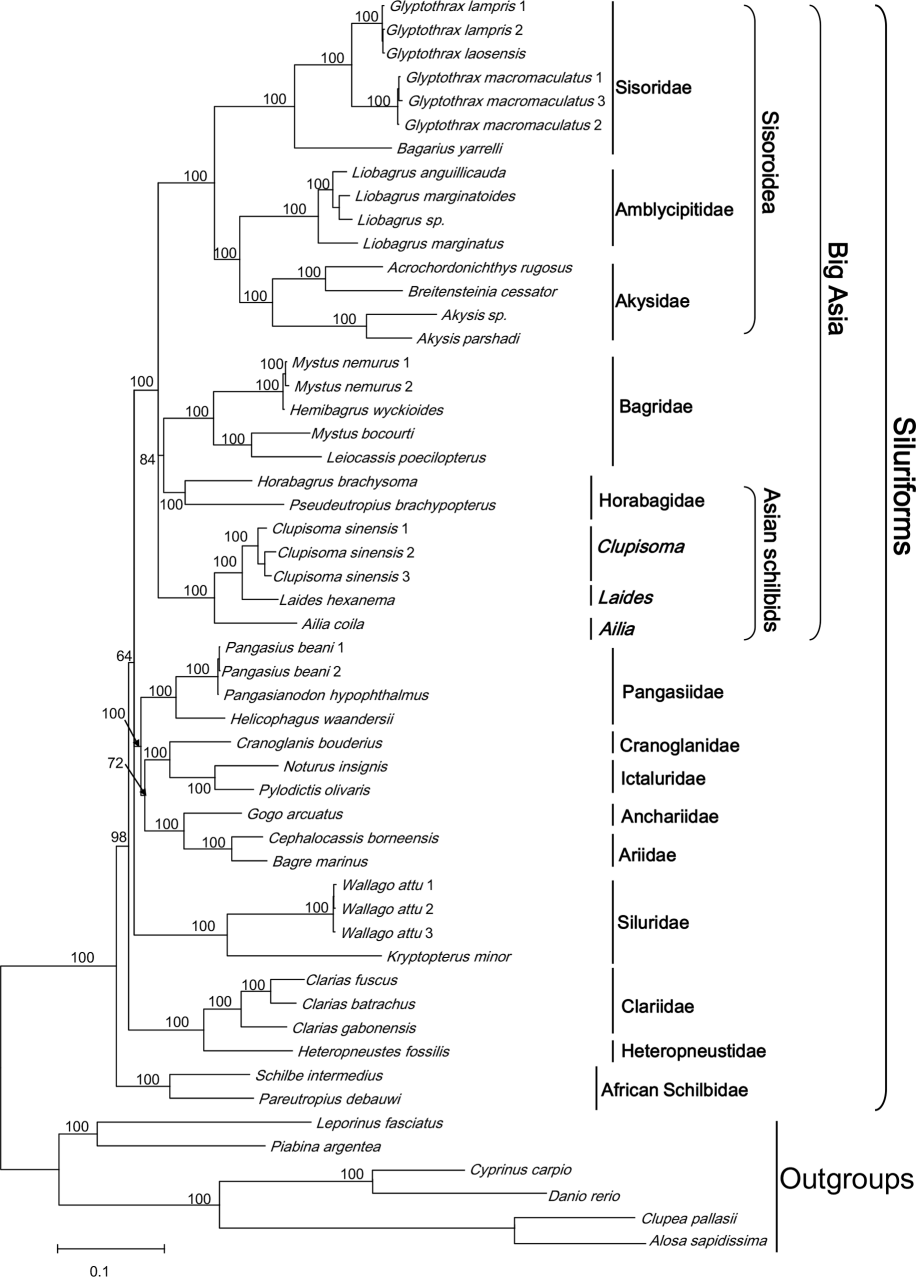
Phylogenetic relationships of the Siluriformes based on a Bayesian inference analysis of concatenated mtDNA genes and partitioned nuclear genes. Nodal support values are Bayesian posterior probabilities. The names Sisoroidea and “Big Asia” are after Sullivan *et al*. (2006) [8].

The obtained nuDNA trees for the analyzed five Asian schilbid genera (Fig 3; ML and MP trees not shown) were somewhat similar to those of the mt genes trees. Analyses of both genomes resolved two strongly supported monophyletic clades: “Big Asia”, i.e., (*Ailia (Laides*, *Clupisoma*)) (BI = 100%, ML = 100% and MP = 100%) and (*Horabagrus*, *Pseudeutropius*) (BI = 100%, ML = 100% and MP = 100%). The genomes differed in that the clade (*Ailia* (*Laides*, *Clupisoma*)) did not associate with other taxa in former “Big Asia”. Further, (*Horabagrus*, *Pseudeutropius*) had a weakly supported relationship with the family Bagridae.

### AU test

The AU test (Table 4) detected significant differences between the mtDNA and nuDNA datasets (P<0.05). Thus, the matrilineal history differed from that of biparental inheritance. We believe this result precluded combining the data sets for phylogenetic analysis inference because each genome had an independent history. However, we retained the result for readers who might be interested in concatenated data results.

**Table 4:** AU test.

	rank	au	bp	kh
mt BI	1	0.811	0.723	0.744
nuclear BI	2	0.271	0.259	0.256
mt+nuclear BI	3	0.023	0.018	0.02

### Expanded dataset of Sullivan *et al*. (2006) [8]

**Fig 4:**
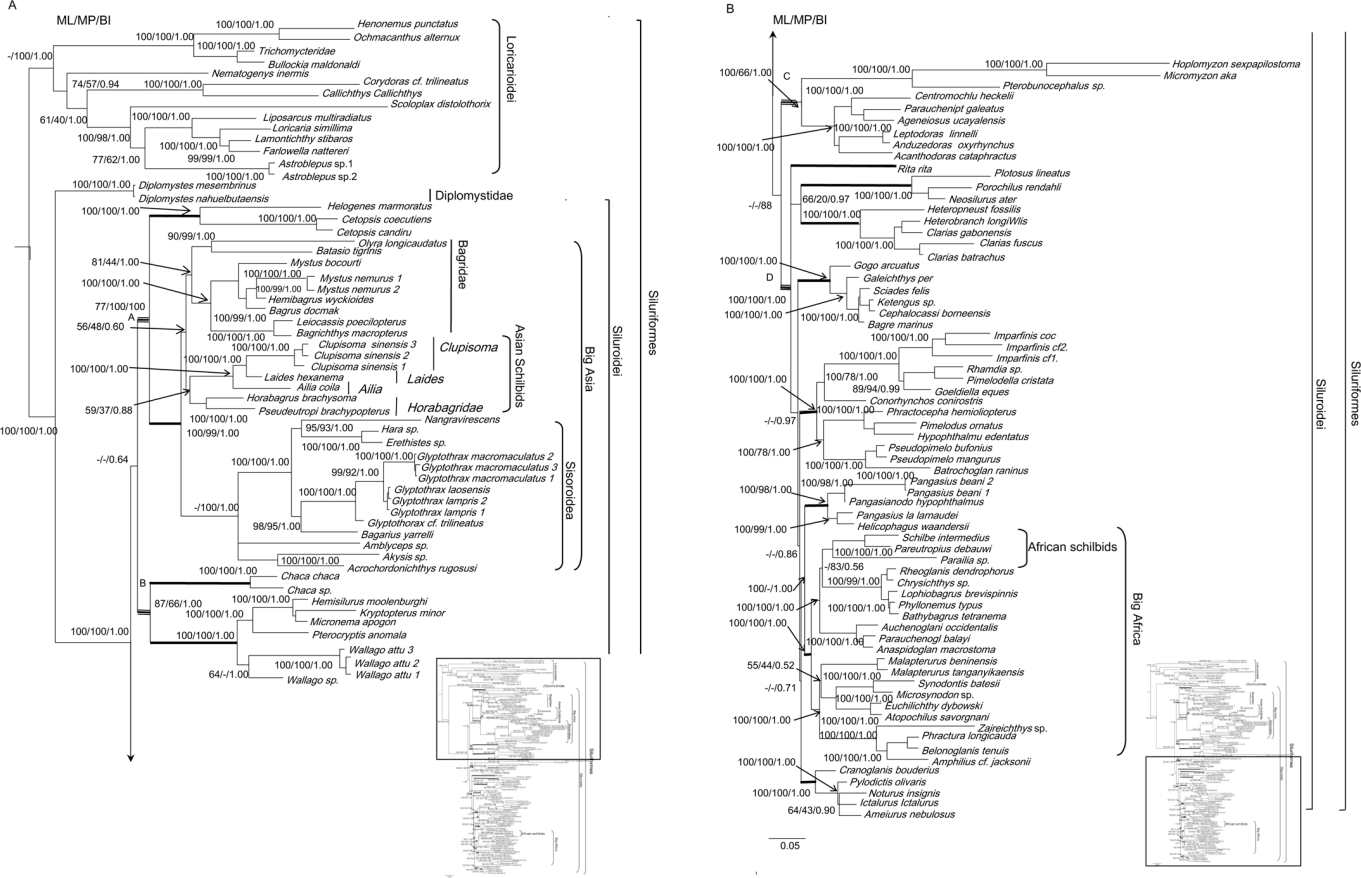
Phylogeny of catfishes based on a dataset expanded from Sullivan *et al* (2006) [8] with nodal support values for BI, ML, and MP, respectively. The 12 lineages marked by thick branches correspond with those revealed by Sullivan *et al*. (2006) [8]. **(A) Part one of phylogeny of catfishes.** The first two clades marked by A, B and ladder-like branch lines are newly resolved herein. **(B) Part two of phylogeny of catfishes.** Nodal support values are indicated on the branches. The last two clades marked by C, D and ladder-like branches are newly resolved herein.

To verify the results from the combined nuDNA dataset, we downloaded the *RAG1* and *RAG2* sequences of Sullivan *et al*. (2006) [8] from Siluriformes, to which we added our de novo sequences (Table 1). We reconstructed the ML, MP and BI trees (Fig 4A and 4B). The five Asian schilbid genera remained a monophyletic group with relationship within “Big Asia” shown as ((*Aailia* (*Laides*, *Clupisoma*)), (*Horabagrus*, *Pseudeutropius*)). However, this arrangement did not enjoy strong support (BI BPP = 0.88, ML BS = 59%, MP BS = 37%). At higher levels within “Big Asia,” the relationships among the genera in the Bagridae, and the superfamily Sisoroidea were poorly resolved.

Analyses of the expanded dataset further resolved relationships within Siluroidei *sensu* Sullivan *et al*. (2006) [8]. Their 13 strongly supported monophyletic linages (thick branches in Fig 4A) were recovered along with the further clustering of these groups into major clades(A, B, C and D (Fig 4B). Sullivan *et al*. (2006) [8] did not obtain interrelationships among their 13 lineages.

## Discussion

### Phylogeny of Asian schilbid genera

Our analyses consistently support both African and Asian schilbids as monophyletic groups, and show that they are distantly related to one another. Thus, we confirm the non-monophyly of the Schilbeidae as recognized by Peng *et al*. (2005) [6], Hardman (2005) [7] and Sullivan *et al*. (2006, 2008) [8, 9].

Recognition of the groups (*Ailia* (*Laides*, *Clupisoma*)) and (*Horabagrus*, *Pseudeutropius*) foes not support the monophyly of the so-called “Big Asia” (Figs 1–4) as proposed by Sullivan *et al*. (2006, 2008) [8, 9]. Analysis of the combined mt gene data and the combined nuclear gene data suggest different suites of relationships among the two groups and other taxa. In the former analysis (Fig 1), the group (*Ailia* (*Laides*, *Clupisoma*)) appears as the sister taxon of the Sisoroidei, and the group (*Horabagrus*, *Pseudeutropius*) is the sister taxon of the Bagridae. In contrast, analyses of the combined nuclear data unite the two groups as sister subgroups (Fig 2). Analyses of the expanded dataset of Sullivan *et al*. (2006) [8] supports this relationship (Fig 4). Because AU testing does not reject either genomic tree, the two results may be equally reliable

Morphological and molecular phylogenetic studies of subsets of the Asian Shilbeidae have been undertaken by Mo (1991) [3], De Pinna (1993) [4], Diogo *et al*. (2004) [5], Peng *et al*. (2005) [6], Hardman (2005) [7] and Sullivan *et al*. (2006, 2008) [8], resulting in differing hypotheses of the relationships among these fishes. This might be in part an artifact of sampling, in particular, the absence of critical taxa. Our study is the first to detail the phylogenetic relationships for all nine recognized genera of Asian schilbids.

In a morphological study, Mo (1991) [3] concluded that the Asian schilbids including *Clupisoma* comprised two distinct groups: *Ailia* and the genera *Horabagrus*, *Pseudeutropius* and *Platytropius*. Our results from mtDNA analyses somewhat supports their result by Mo (1991) [3] did not clearly comment on the relationships of *Clupisoma* or specify which species of *Platytropius* were examined. He claimed *Ailia* was associated with the Clariidae and Heteropneustidae while *Horabagrus*, *Pseudeutropius* and *Platytropius* were closer to the Bagridae and Pangasiidae, which differs from our results. We did not have access to De Pinna’s (1993) [4] unpublished dissertation. Thus, we do not know if he examined *Clupisoma*. Researchers citing his dissertation state that he assigned *Horabagrus* to its own family because it was distinct from both the Schilbeidae and Bagridae [8]. Further, De Pinna (1993) [4] proposed that all schilbids (including African species) constituted a monophyletic group with the subgroup (Schilbinae (Ailiinae, *Laides*) being closer to the Pangasiidae than to the Shibeidae (see Fig 2 of Hardman, 2005) [7]. In contrast to our findings, and using a less complete set of Asian schilbids than included in the present study, De Pinna concluded that the Shilbeidae was monophyletic. Diogo *et al*. (2004) [5] examined Asian *Ailia*, *Laides* and *Pseudeutropius*, and African *Schilbe* and *Siluranodon*, and similar to De Pinna obtained results that differed from ours, concluding that the Schilbeidae exclusive of *Horabagrus* was monophyletic and its sister-group was the Pangasiidae. Unlike Pinna (1993) [4], Diogo *et al*. (2004) [4] did not propose intergeneric relationships among *Ailia*, *Clupisoma*, *Horabagrus*, *Laides* and *Platytropius*.

The molecular phylogenetic studies of Peng *et al*. (2005) [6] failed to resolve the relationships of Asian schilbids because they sampled Asian *Clupisoma* only, although they suggested that Chinese schilbids might be closest to either the Bagridae or Siluridae. Hardman (2005) [7] resolved the relationships as (*Pseudeutropius* (*Horabagrus*, *Clupisoma*)) and assigned these genera to the Horabagridae created by De Pinna. However, owing to absence of *Ailia* and *Laides*, his study failed to provide an overall phylogenetic scenario of the five genera of Asian schilbids. Further, his resolution of the relationships of *Clupisoma* differed from ours.

Sullivan *et al*. (2006, 2008) [8, 9] clustered *Ailia* with *Ladies*, and *Horabagrus* with *Pseudeutropius* with strong support. Both groups belonged to “Big Asia.” The group (*Ailia*, *Laides*) was weakly placed as the sister taxon of the Sisoroidea and the group (*Horabagrus*, *Pseudeutropius*) was weakly supported as the sister taxon of Bagridae in their MP and ML trees. Thus, their results are similar to ours based on mtDNA analyses. They could not place *Clupisoma* owing to its absence in their analyses.

In summary, we propose that 1) the group (*Ailia* (*Laides*, *Clupisoma*)) is monophyletic and 2) its sister-group, based on nuDNA analyses, appears to be (*Horabagrus*, *Pseudeutropius*), although this hypothesis conflicts with the matrilineal genealogy based on mtDNA data. Our work specifies the phylogenetic position of *Clupisoma*, which heretofore was ambiguous, and our hypothesis differs from that of Hardman, which Sullivan *et al*. (2006, 2008) [8, 9] assumed to be true.

### Tree sensitivity

Many factors affect the topologies of phylogenetic trees, including choice of outgroup, ingroup representation, the evolution of genes, long-branch attraction (LBA), and method of tree construction [27]. Two of these factors considerably affect the topologies of the trees for catfishes: choice of genome and taxonomic representation. Phylogenetic relationships based on the mtDNA and nuDNA differ significantly, a discovery termed cytonuclear discordance [28]. The resulting trees differ not only among the members of “Big Asia” but also among other catfishes (Figs 1 and 2). The conflict is not unusual [29, 30]. Our results reinforce the hypothesis that nuclear and mt genes may have different evolutionary trajectories.

The density of ingroup sampling also affects trees. The addition of 17 ingroup sequences (Table 1) to the dataset of Sullivan *et al*. (2006) [8] changes the topology of the tree greatly. It further resolves the relationships among the 13 lineages comprising the suborder Sisoroidei (Fig 4). Saitoh *et al*. (2006) [31], Wang *et al*. (2007) [32], Li *et al*. (2008) [33], Yang *et al*. (2010) [34], Telford and Copley (2011) [27] and Wang *et al*. [35] emphasized the importance of increasing the density of ingroup sampling. The present study provides support for this approach.

### Taxonomic implications

Taxonomy should reflect historical relationships [36]. Based on his own analyses and those of Mo (1991) [3], Hardman (2005) [7] recognized the Horabagridae of De Pinna (1993) [4] as containing the genera *Horabagrus*, *Pseudeutropius* and *Clupisoma*. Sullivan *et al*. (2006) [8] followed this assignment. Our results support the recognition of the Horabagridae vis-à-vis Asian taxa, but with the exclusion of *Clupisoma*. The Horabagridae De Pinna (1993) [4] contains *Horabagrus* and *Pseudeutropius* only. We note that sometimes *Horabagrus* has been assigned to the Bagridae [3].

Recognition of the Horabagridae renders the Schilbeidae a polyphyletic family. The type genus of Schilbeidae, *Schilbe*, is native to Africa. Because African schilbids are not the sister group of Asian genera [3], and to obtain a taxonomy that reflects the phyletic history of these Asian catfishes, we formally erect a new family Ailiidae fam. nov. (type genus *Ailia*) for monophyletic Asian group comprised of the genera *Ailia*, *Laides* and *Clupisoma*. This results in recognition of the following taxonomy for these catfishes:

Class Actinopterygii

Order Siluriformes

Suborder Sisoroidei

Family Horabagridae: *Horabagrus* (Asia), *Pseudeutropius* (Asia)

Family Ailiidae fam. nov.: *Ailia* (Asia), *Laides* (Asia), *Clupisoma* (Asia)

Family Schilbeidae: *Schilbe* (Africa), *Irvineia* (Africa), *Pareutropuis* (Africa), *Parailia* (Africa), *Siluranodon* (Africa), *Platytropius* (Asia), *Eutropiichthys* (Asia),*Neotropius* (Asia), *Proeutropiichthys* (Asia), *Silonia* (Asia) [3]

We do not have any specimens of Horabagridae or Ailiidae, we obtained the morphological information of seven species within these two lineages from FishBase (http://www.fishbase.org/search.php?lang=English). Unfortunately, only one morphological trait was available for all seven species. The total numbers of soft rays of anal fin in Horabagridae ranged from 31 to 33, while the ones in Ailiidae ranged from 39 to 55 (S1 Table). These data are congruent with our hypothesis of a new family of Ailiidae. In addition, images displayed on the website show differences in body shape: the abdominal line of Horabagridae tends to be flat, while those of the Ailiidae curve. These data also show divergence between these two lineages (S1 Fig). The morphological differences correspond with the molecular evidence for a new family.

Undoubtedly, morphological evidence is crucial to propose a new family from within an established family. We encourage the acquisition of deeper morphology evidence or other disciplines to further test our hypothesis of the Ailiidae.

### Accession Numbers

All the sequences by this study have been submitted to GenBank. The accession numbers together with the downloaded data were listed in Table 1.

## Supporting Information

S1 Fig Two species of Horabagridae and five species of Sisoroidei. (PDF)

S1 Table Comparisons of counts of anal fin(s) within the seven species of Horabagridae and Ailiidae. (DOCX)
