# Supplementary material for: Phylogenetic Relationships of Five Asian Schilbid Genera Including Clupisoma (Siluriformes: Schilbeidae)
Source: PLoS One. 2016 Jan 11;11(1):e0145675. doi: 10.1371/journal.pone.0145675 (PMC4713424; doi:10.1371/journal.pone.0145675)

S1 Fig. (a) *Horabagrus melanosoma* (b) *Pseudeutropius indigens* (c) *Clupisoma sinense* (d) *Clupisoma roosae* (e) *Clupisoma prateri* (f) *Laides hexanema* (g) *Laides longibarbis*

All images adopted from FishBase (http://www.fishbase.org/search.php?lang=English).

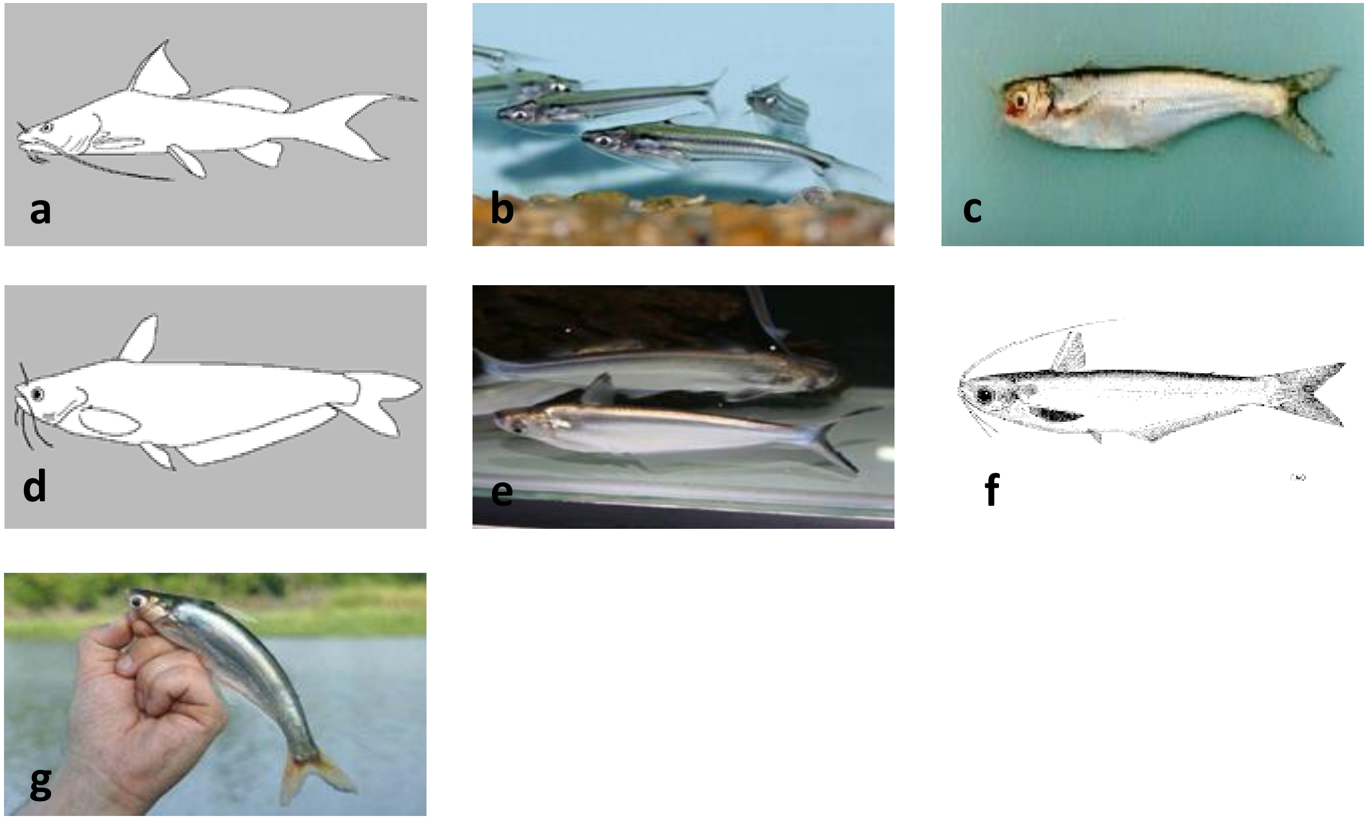

Supplement: S1 Fig — (PDF) [file pone.0145675.s001.pdf]
