# Supplementary material for: Phylogenetic Relationships of Five Asian Schilbid Genera Including Clupisoma (Siluriformes: Schilbeidae)
Source: PLoS One. 2016 Jan 11;11(1):e0145675. doi: 10.1371/journal.pone.0145675 (PMC4713424; doi:10.1371/journal.pone.0145675)
Supplement: S1 Table — (DOCX) [file pone.0145675.s002.docx]

## S1 Table. Comparisons of counts of anal fin(s) within the seven species of Horabagridae and Ailiidae

|  | **Horabagridae** | | **Ailiidae** | | | | |
| --- | --- | --- | --- | --- | --- | --- | --- |
|  | [***Horabagrus melanosoma***](http://www.fishbase.org/summary/SpeciesSummary.php?genusname=Horabagrus&speciesname=melanosoma) | [***Pseudeutropius indigens***](http://www.fishbase.se/summary/SpeciesSummary.php?genusname=Pseudeutropius&speciesname=indigens) | [***Clupisoma sinense***](http://www.fishbase.org/summary/SpeciesSummary.php?genusname=Clupisoma&speciesname=sinense) | [***Clupisoma roosae***](http://www.fishbase.org/summary/SpeciesSummary.php?genusname=Clupisoma&speciesname=roosae) | [***Clupisoma prateri***](http://www.fishbase.org/summary/SpeciesSummary.php?genusname=Clupisoma&speciesname=prateri) | [***Laides***](http://researcharchive.calacademy.org/research/ichthyology/catalog/fishcatget.asp?genid=9064)  [***hexanema***](http://researcharchive.calacademy.org/research/ichthyology/catalog/fishcatget.asp?spid=5619) | [***Laides longibarbis***](http://www.fishbase.org/summary/SpeciesSummary.php?genusname=Laides&speciesname=longibarbis) |
| **Fins number** |  |  |  | 1 | 1 |  | 1 |
| **Spines total** |  |  |  | 0-0 | 0-0 |  | 0-0 |
| **Soft-rays total** | 31 - 33 | 31 - 33 | 44 - 50 | 47-55 | 41-46 | 39 - 42 | 41-47 |

Note: We obtained some morphological data for seven species within two lineages (Horabagridae and Ailiidae) from FishBase (http://www.fishbase.org/search.php?lang=English). Unfortunately, as the limited evidence of morphological data, only one morphological trait that anal fin(s) was involved in all seven species, so we just put this table of anal fin(s) data in our supporting information. Other morphological data detailed on the website.
